# Municipalities Collaborating in Public Health: The Danish Smoking Prevention and Cessation Partnership

**DOI:** 10.3390/ijerph7113954

**Published:** 2010-11-09

**Authors:** Pernille Tanggaard Andersen, Walid El Ansari, Hanna Barbara Rasmussen, Christiane Stock

**Affiliations:** 1 Unit for Health Promotion Research, Institute of Public Health, University of Southern Denmark, Niels Bohrs Vej 9-10, 6700 Esbjerg, Denmark; E-Mails: ptandersen@health.sdu.dk (P.T.A.); cstock@health.sdu.dk (C.S.); 2 Faculty of Applied Sciences, University of Gloucestershire, Oxstalls Campus, Oxstalls Lane, Gloucester GL2 9HW, UK; 3 Centre of Maritime Health and Safety, University of Southern Denmark, Niels Bohrs Vej 9-10, 6700 Esbjerg, Denmark; E-Mail: hbrasmussen@cmss.sdu.dk

**Keywords:** partnership, coalition, smoking cessation, Denmark, multi-site evaluation, health professions education, leadership

## Abstract

This study explored the Smoking Prevention and Cessation Partnership (SPCP) which builds upon a collaboration between two Danish municipalities targeted at the prevention of tobacco smoking. The aim of the study was to describe the processes of SPCP, to examine the difficulties this collaboration faced, and to assess how these experiences could be used to improve future partnership collaboration. We employed qualitative methodology comprising 12 semi-structured one-to-one interviews with SPCP’s stakeholders and an analysis of the partnership documents and reports. The findings suggested that the main potentials of the partnership were the personal relations between the members and stakeholders with the possibilities of the creation of new connections with other actors. Barriers to successful partnership building were the implementation of the new Local Government Reform as a competing task, and that the two municipalities were heterogenic in respect to organizational issues and working methods. Other impediments included the lack of continuity in leadership, the lack of clarity regarding the form of collaboration and roles, as well as different expectations of the stakeholders. We conclude that four factors remain critical for partnerships. The first is the clarity of the collaborative effort. Second, partnerships need to take into account the structural circumstances and culture/value systems of all stakeholders. Third is the impact of contextual factors on the development of the partnership; and the fourth factor is the bearing of personal/individual factors on the partnership e.g., personal engagement in the project. Early attention to these four factors could contribute to more effective partnership working.

## 1. Introduction

In Denmark, the percentage of adults who reported that they smoked everyday has decreased by more than half, declining from 47% in 1984 to 23% in 2008 [1]. However, although current smoking rates among adults in Denmark are around the same as the Organization for Economic Co-operation and Development countries’ average, they are still higher than in other Scandinavian countries [1]. In many countries, tobacco control and smoking cessation efforts have employed multi-stakeholder partnership arrangements for smoking cessation, as the recent decades witnessed partnerships being increasingly initiated and utilized to address a wide range of public health challenges [2,3]. Hence, collaborative projects that address tobacco consumption have been instigated between countries or researchers [4], sometimes employing community-primary care-academic partnership models [5]. In line with the Ottawa Charter for Health Promotion which calls for strengthening community action and for coordinated intersectoral activities [6], such joint working builds upon collaborative approaches in order to tackle public health dilemmas [7]. In such inter-agency arrangements, partners join forces to engage in collective health objectives and outcomes [8]. The term ‘partnership’ denotes associations of institutional and community agencies that joined forces to address common health concerns and communal outcomes [9]. Similarly, ‘collaboration’ is to work jointly with others, where those collaborating take on specified tasks within the project and share responsibility for its success. In this paper, the terms ‘partnership’ and ‘collaboration’ are used interchangeably.

Partnership arrangements have been examined in many individual countries. For instance joint working efforts have been evaluated in the USA [10]; in the UK; and in South Africa (e.g., [1]). The World Health Organization has also commissioned several appraisals of coalitions across several countries [11,12]. However, whilst some evaluations have been carried out in some Nordic countries (e.g., [13–15]), to the best of our knowledge, very little research on partnership working has been undertaken in some countries e.g., in Denmark. To this end, the present study assessed the value of partnership working within the Danish context, as well as the associated barriers and challenges that are encountered when municipalities collaborate together.

### 1.1. The Danish Experience

In 2002 the liberal government of Denmark established a commission for local government reforms in Denmark, which completed its work in 2004 [16]. Meanwhile in 2005 the new Danish Health Law established a new structure for local governments. In 2007 these local government reforms became effective leading to the creation of new structures and geographically new ‘regions’ that consisted of bigger municipalities comprised of previously smaller geographical areas. Consequently the reforms also shifted responsibilities and influenced the relationships between the three tiers of the Danish political administration system: the macro level (State, central); the meso level (counties and later regions); and the micro level (municipalities).

Table 1 depicts the government reforms in Denmark and the accompanying changes in duties and tasks. The reforms reduced the actions that were undertaken by the meso level (previously termed ‘counties’ now ‘regions’), as important functions in all policy areas had been moved to either the central or municipal levels. For health sector activities, the regions still maintained the responsibility for somatic and psychiatric hospitals and their development, whereas former functions (e.g., chronic diseases and health promotion) were reduced and transferred either to the municipal or state levels. The rationale behind these changes was that due to the larger size of the newly-formed regions and their expected higher competences, the regions would now be better able to plan hospital restructuring, and to deliver enhanced quality and efficient hospital management. Within health and health care, the tasks of the larger municipalities were also extended, particularly in the field of prevention and health promotion. In the meantime at the central level, the supervising and regulating roles of state bodies (e.g., National Board of Health) were further strengthened. Collectively, these structural changes and the prevention tasks that accompanied the changes form the basis of understanding why inter-municipal collaboration became more important and imperative in this new Danish context.

### 1.2. The Smoking Prevention and Cessation Partnership (SPCP)

In 2006, all municipalities in Denmark had an opportunity to apply for funding from The National Board of Health for developing and maintaining inter-municipality collaborative initiatives aimed at smoking prevention and cessation. Two municipalities in the west of Denmark (Varde and Esbjerg) applied together, and in 2007, the Danish SPCP was created as a collaborative model. SPCP’s stakeholders included health professionals and general practitioners, health care units, pharmacies, midwife centres, the health assistant education programme, outreach/public health nurses, citizens with chronic conditions, along with other partners such as private enterprises/businesses (e.g., a packaging company) and other health or educational agencies (e.g., patient associations and a primary school). The purpose of this inter-municipal SPCP was to strengthen the health promotion efforts of both municipalities, particularly focussing on the prevention of tobacco smoking, and the improvement in implementation of smoking interventions through partnership working and exchange of experience. The SPCP was aimed at: (1) activities preventing the use of tobacco whilst motivating citizens not to start smoking (e.g., through school-based health promotion and educational activities); (2) smoke-free environments; and (3) courses in quitting tobacco smoking (see below). However, during the course of the partnership, a new Governmental Act was instated that prohibited smoking in working places and on the job, in institutions and schools, in other educational institutions, on collective/communal means of transportation and in most restaurants (Act no. 512. Ad 6. June 2007). Hence, the implementation of this Act led to a shift in the aims of the partnership towards a focus predominantly on smoking cessation activities. SPCP’s objectives and criteria of success included: increased dialogue between both municipalities; joint working on non-smoking policies in private-sector work places; and, the gaining of experience in relation to municipal-level prevention of tobacco use. As such, SPCP was seen as the foundation for expanded future collaborations between both municipalities. This paper describes the evaluation of this inter-municipal “Smoking Prevention and Cessation” partnership.

SPCP’s activities included courses in smoking cessation that were undertaken in almost all pharmacies in Denmark and were offered to either individual smokers or to groups of smokers. Beginning with the individual smoker’s needs, the pharmacy provided advice on smoking cessation. Each smoking cessation course span over 4–10 weeks duration and comprised five sessions of counselling, each about 1.5 to 3 hours. These sessions included testing of blood nicotine levels, measurement of blood and exhaled carbon monoxide levels, advice on smoking cessation and on choice and utilisation of nicotine replacements, in addition to scheduling a follow-up in order to document whether participants were progressing in the period of the smoking cessation. The sessions also involved support and advice to the smoker that further focussed on the effects of environmental tobacco smoke on colleagues at work or for the smoker’s family. At the end of any given course, the smoker’s support and future needs were discussed and agreed upon, and follow-up was established as to whether the smoker wished to be phone-contacted by the smoking cessation consultants 6 months later. Across Denmark, all the smoking cessation courses followed the same pattern with some minor variations, and in all cases the smoker had to pay the pharmacy for the course.

### 1.3. Aim of the Study

The study was conducted in collaboration with two Danish municipalities. The purpose of the evaluation was to describe the processes of SPCP, to examine the difficulties that this collaboration faced and the reasons behind the difficulties, and to assess how these experiences could be used to improve future partnership collaboration in Denmark and elsewhere. The four specific objectives were to:

- describe the collaboration of the partnership’s stakeholders;
- assess the barriers, challenges and expectations among the various stakeholders;
- provide recommendations on how the partnership could be further developed to advance the aims of the SPCP; and,
- appraise whether such ‘obligatory’ collaboration between municipalities in reference to smoking prevention is at all feasible or desirable.

### 1.4. Conceptual Framework

A partnership is a formal collaboration between organisations, groups and agencies to reach a common goal. Guided by the published literature on partnerships, and in order to steer the evaluation, we employed the conceptual model published by El Ansari & Phillips [17]. The model is informed by coalition theories that address membership, resources, support, processes, functions, and roles [18]. It also explores many features comprising personnel factors and barriers; organizational factors and barriers; power-related factors; time factors; and outcomes. Such characteristics enable partnerships to fulfill their goals. [19]. Hence, the model considers the game theorists’ emphasis on a coalition’s payoffs (outcomes); the social psychologists’ emphasis on resources; and the political scientists’ emphasis on importance of ideology similarity in partnerships [20]. Technically, the model provides an appraisal framework that enhances learning through evaluation, while simultaneously ensuring that partnership processes were not overlooked. The model also deals with coalition barriers of organization, of attitude, of vision and of ignorance [21]. Operationally, the model’s strength is its comprehensiveness, and its adaptability and applicability to multi-stakeholder initiatives in different contexts [17]. Hence it was felt appropriate to employ it in the analysis of partnership approaches in the Danish setting.

## 2. Methods

### 2.1. Sample, Ethics and Data Collection Procedures

The study collected information about the details and development of a collaborative effort between two municipalities, in addition to the collaboration between the municipalities and a private company and pharmacies that participated in implementing the smoking cessation courses. We employed a qualitative approach because the aim was to undertake an in-depth study of the processes of collaboration, the difficulties and promises associated with such collaborative efforts, and to assess how the participants’ experiences could be applied in order to enhance the efficiency of future partnership working. Hence the present study utilised individual (stakeholder) interviews. The interviews comprised in-depth conversations in order to gather qualitative information and the views of those individuals involved in a particular initiative or project, its context, implementation, results and impact. Such interviews can provide feedback on all aspects of a project’s inputs, activities, outputs, outcomes. Hence the research team employed this technique in order to: elicit the preliminary views of stakeholders; emphasize the individual *versus* group concerns; and explore any divergent experiences and ‘outlier’ attitudes [22]. The research was approved by both municipalities and the University of Southern Denmark, and potential interviewees were contacted and informed that by accepting to be interviewed they were providing their verbal informed consents. Participation was voluntary and data protection was observed throughout study.

The first and third authors undertook 12 semi-structured one-to-one interviews with a range of strategic SPCP stakeholders e.g., health consultants, social workers and project leaders in the municipalities. These individual interviews included members of SPCP’s steering group (n = 2); former and present SPCP leaders (n = 2); other partners involved in the SPCP in both municipalities (n = 1); and smoking cessation consultants from both municipalities (n = 7). In line with El Ansari & Phillips [23], the interviewees were selected to reflect diverse strategic administrative cadres and functional positions in the SPCP. Each interview lasted 45–90 minutes. A topic guide led the direction of inquiry. Interviews were audio-recorded, and the interview questions asked respondents about their concerns regarding the positive (conducive) aspects and negative (barriers) features of SPCP, in addition to the stakeholders’ expectations, and SPCP’s processes. Additionally, the research team undertook a minor documentary analysis on SPCP’s published information.

### 2.2. Data Analysis

The audiotapes were reviewed by the authors, and the data were transcribed and analyzed in the text using elements from grounded theory and the constant comparative technique [24]. In the coding process we moved from open coding (word-by-word) to axial coding (categories were connected with subcategories) [25]. The transcribed text was read/re-read and partnership-relevant items were identified and indexed to headings and categories (validated in later readings). Initial coding produced provisional concepts, and codes were compared and clustered into categories, then matched for links [24]. Analysis was staged: (1) data review; (2) data inclusion; (3) cluster formation; and (4) review, with sense making and finding representations. The emerging themes were backed by quotations for descriptive immediacy. The first and third authors analysed the interviews while the second and fourth authors provided a peer-review of the analysis. An audit trail ensured quotes were traceable to coded participants but ensured confidentiality [26].

In addition the research team undertook a minor documentary analysis on the published information that was provided by the SPCP: partnership documents, pamphlets, brochures, statistics, evaluation reports and other information were all analysed for their content. The documentary analysis was conducted to achieve a contextual understanding of the policy and practice environment in the municipalities. In the documentary analysis, we systematically analyzed the content, the motivation, intent and purpose of documents employing content analysis as a technique [27,28]. The documentary analysis was useful in providing information about the organisation, values, political mandate and task, in addition to creating an understanding of the context for the collaborative effort. Thus, it provided a useful background picture that enhanced the analysis of the interviews [29].

## 3. Results

The data collection process was not straight forward as it was challenging to precisely identify the organisational participants who were actually actively participating in the SPCP. As with other partnerships elsewhere [30], membership sign-up or termination procedures are usually lacking: this poses difficulties in determining who is actually a current partnership member. Further, because of a change of SPCP’s leadership, data about the smokers who participated in the smoking cessation course and those who were trained as smoking cessation consultants were not available to the evaluation team. Thus, we were only able to contact half of the smoking cessation consultants.

### 3.1. Outcomes: Smoking Cessation Rates

Analysis of the partnership documents and reports indicated that since January 2007 Esbjerg and Varde municipalities collected various statistics on the characteristics of SPCP participants. These included socio-demographic features (e.g., gender, age, employment status, home ownership) and other aspects related to smoking (smoking status, number of years as a smoker), along with the number of cessation courses that were administered in both municipalities. Between 2007 and 2008, 213 smokers participated in the smoking cessation courses. Males comprised 43% of the participants, 85% were ≥45 years of age, 75% were employed and 70% were home owners.

As regards the impact of the smoking cessation course, 66% (n = 141) of participants quitted smoking at the end of their course (smoking cessation status was confirmed by participants but not bio-chemically validated). Of those participants who quitted smoking at the end of their course, 43% maintained their smoking cessation for 6 months after the course. Chi-square (χ^2^) tests showed that factors significantly associated with quitting smoking after completing the course were older age (*p* = 0.03) and a longer duration of smoking (*p* = 0.002), while other factors like gender or employment status were not associated with quitting smoking.

### 3.2. Process: Partnership Working and Opportunities for Improvement

SPCP focussed on smoking prevention and cessation activities and the exchange of experience as an initial step for future collaborative health promotion efforts. SPCP felt that a common leader would strengthen the partnership between the two municipalities, hence a project leader was recruited and was stationed in Esbjerg municipality but visited Varde municipality regularly. Given that the partnership was initiated shortly after the Danish municipality reforms described above, there were redeployments of personnel in the health promotion departments between both municipalities.

Five main themes emerged from the analysis of the interviews: real partnership, clarity, culture, leadership, and relationships. In the next section we provide examples of the challenges to progress that were encountered, supported by interviewees’ quotes. The underlying impediments are outlined.

### 3.3. Real Partnership?

The partnership between the two municipalities could have functioned better. For instance, in the planning phase there were intentions and expectations about a *real* partnership, and it was not foreseen that the national reforms would consume considerable time and resources of the municipalities. This meant that SPCP was a lower priority than the reforms that were simultaneously put into operation:

“We have definitely underestimated the municipality reform. And, oh, we had much bigger expectations to being able to cooperate. If we had known how, looking back, so we can see that it was completely unrealistic” (respondent 1).

Hence ‘external’ organisational factors influenced the setting up, initiation and actual implementation of SPCP. The reforms had substantial influence on duties and responsibilities that in reality it could have acted as a barrier to realising a real partnership between the municipalities. The result was two separate projects rather than one *real* partnership:

“You can’t say that is was a cooperation project, there are two projects; one project, but in practice we run them separately, we had some intentions about how we as municipalities, via this project and as neighbouring municipalities, could find some common cooperation norms, and we could transfer it to other activities, but we won’t get that, we won’t get that out of it” (respondent 1).

Meanwhile, the smoking cessation consultants had high expectations about common collaborative smoking cessation activities that span the borders of both municipalities. However, such truly collaborative actions never came to light. This further draws attention to the context in which a partnership is being initiated and put into practice, as well as its timing in relation to other activities that are concurrently happening nationally or locally.

### 3.4. Clarity

Other challenges were the lack of clarity from the inception as to the extent of the cooperation, and hence the stakeholders’ expectations and roles:

“From the beginning it should have been stated at which level the cooperation should be. Is it cooperation at the employees’ level, at leadership level or at a national level? Is it development or implementation? Or where do they wish to have cooperation?” (respondent 3).

Collaboration requires much clarity, transparency and time. Such lack of clarity was questioned:

“What is it they want with it and what effort will be put in this cooperation? Because cooperation takes time, it means that you have to give high priority to the employees who are involved in the partnership and give them permission to use time on it” (respondent 8).

Conflicting messages contribute to lack of clarity. For instance, the smoking cessation consultants were trained in smoking cessation through SPCP, but their departmental leaders subsequently refused that the consultants use their time to conduct the courses:

“Many of them, who were actually taking a course, got a message from their leaders, that they shouldn’t conduct smoking cessation courses. There were some restrictions about what they were allowed to do and what they were not allowed to do” (respondent 7).

### 3.5. ‘Culture’ and ‘Ways of Doing Business’ of the Participating Agencies

The two municipalities had different ‘ways of doing business’ that reflected the unique cultures of the distinctive agencies that participated in SPCP:

“In Varde we have a general attitude: do something and then get forgiveness for it later, hence we start some activities and formalize them later. In Esbjerg all has to be formalized before starting and when everything is ok you can start up the activities, so it has been different workings methods” (respondent 1).

As an administration, Esbjerg municipality is bigger than Varde, and exhibited more hierarchical structures. The result was that for SPCP planning, some decision-making required relatively more time in Esbjerg than in Varde:

“It was very difficult to cooperate internally in Esbjerg municipality, because of their hierarchy, it was quite difficult to come through to those you should talk with and get answers and permission to do what you were supposed to do. So I think that in Esbjerg it was very difficult and it went very slowly, there is a lot of hierarchy, but in Varde it was much easier….I found out later that there was much more willingness and it was much easier to get through with the project in Varde, there was much more willingness, interest, there was not the same hierarchy to fight with” (respondent 2).

Each municipality represented a political organisation and the priority of different areas within health ultimately depended on the leadership and politicians who would support the proposed areas of work. Such political-level decisions were evident in both municipalities and influenced SPCP’s activities. For example, in Varde a Health Centre was created to lead on the health promotion activities and hence SPCP activities. Conversely in Esbjerg, the Health Department at the municipality led the health promotion activities and therefore also the SPCP activities. However, such differences and variations could serve as valuable opportunities for mutual and cross-learning:

“If we found a way, where the things are going very well in Varde, so of course we should learn from it in Esbjerg, but the problem is that it is working so different in Varde, that we can’t use in Esbjerg and vice versa” (respondent 4).

Differences in stakeholders’ ways of working are not uncommon in partnerships. This was highlighted by an interviewee:

“I don’t think that cooperation between Varde and Esbjerg will be in the near future, but Esbjerg can do it with another municipality and Varde with another, but those two municipalities are too different” (respondent 9).

Hence, the impending structural conditions that were simultaneously implemented nationally could have posed challenges for both municipalities. Further, the working methods of the two municipalities were quite different:

“Everything that could go wrong went wrong in this project, but I don’t think we could predict it, I don’t think so ... the structural reform that aggregated different municipalities in bigger units was a challenge ... it was a big change and it slowed down many of the decisions. And that two municipalities working together with so different working methods plus that we in the steering group have changed places, was also a challenge, but I’m happy that we keep up and didn’t give up” (respondent 1).

### 3.6. Leadership

Partnerships comprise many stakeholders who might not have traditionally worked together. In such joint initiatives effective leadership plays a pivotal role in communicating the vision of the partnership to the partners:

“The first project leader was good to tell us about the project, I think it was really good. We have been told about the organization and we got all the project description. There were a lot of details in the project” (respondent 6).

Successful partnerships are associated with good leadership. However, SPCP’s first leader subsequently left the partnership in order to take up another post and was replaced by another leader:

“It didn’t work so well with the other project leader. I think people don’t burn the same way for this kind of project. I even don’t know if the new project leader wished to be the leader of the project or was pushed in it” (respondent 10).

Interviewees commented that a continuity of the leadership was required and could have benefited SPCP:

“The leaders of health department got sick and then resigned, the leadership in the department was missing and it was me who should carry out the collaboration” (respondent 2).

“There were some meeting (network meetings for consultants) where we were both from Esbjerg and Varde, but then it happened that project leader is leaving, got another job....it happens, and then the new one is coming, but there was so long time without the project leader and I think that is was one of the reason that it didn’t work” (respondent 6).

The continuity of the leadership in collaborative efforts is vital, as during periods when there was no project leader, there was no formalised cooperation between the two municipalities:

“The weakness of project was that our “old” project leader has stopped” (respondent 6).

“The leadership… I say it didn’t work, I don’t think it has something to do with the new project leader. It just went wrong when the first project leader stopped and it was a period of half year without anyone” (respondent 4).

### 3.7. Relationships, Links and New Connections

Partnership working effectively builds on the personal relations between individuals, stakeholders and members of the steering group. Such connections that contribute to garner trust, rapport and relationships were reported by interviewees:

“Cooperation is a lot of personal relations, not that you see each other privately, but that you know each other a little bit and have trust to each other. This is what carries it out” (respondent 3).

Without the mutual relationships and links, it would have been taxing to complete the SPCP, particularly given the multiple internal and external challenges associated with the national reforms:

“The strong sides were the personal relations and the engagement which has been in the steering group and in the project leadership to accomplish the project in spite of all the odds we had, we will have something out of it; that was really good” (respondent 1).

Another feature of the partnership was the possibilities to establish new connections with ‘new’ actors, which would not have been possible without the stakeholders working together as partners. For instance, as a direct result of SPCP, the municipalities forged contacts with agencies that had not previously been in direct links with the municipalities. These ‘new’ links included an independent boarding school for secondary students and one factory:

“The project gave us possibility to have contact with an independent boarding school for lower secondary students” (respondent 5).

“We got some economic help for some things, and to come out to the enterprises and do something, I don’t think we would have done it without the partnership” (respondent 5).

Table 2 summarizes some of the challenges of the Danish SPCP and the barriers that were encountered in its initiation and implementation.

## 4. Discussion

Health and social care agencies are increasingly sharing information and resources with local authorities and governments in order to provide services that meet the needs of their populations [31]. Such inter-organizational relationships include public, nonprofit, and commercial sectors. However, when diverse sectors collaborate together, the barriers of partnership working materialize and could threaten success. All stakeholders need to focus their timely attention to such challenges.

As regards our first objective, we described the collaboration of SPCP’s stakeholders (Table 2). This Danish SPCP delivered smoking cessation courses as a collaborative effort between many agencies, administrators, health professionals, ‘smoking consultants’, pharmacies and citizens from two municipalities. SPCP motivated people not to smoke in the first place, and assisted them in quitting if they were smokers.

However, about 6–8 months after SPCP was initiated, SPCP’s leader accepted another post elsewhere in Denmark. This resulted in a ‘quiet’ period (4–5 months) until a new project leader was appointed. In addition, the two municipalities had different organisational structures and working methods, which further delayed SPCP’s activities. Such procedural delays and/or financial constraints are obstacles for partnership working [17]. Collectively, these factors that influenced SPCP’s relationships and activities are in agreement with the conceptual model [17] we employed for the evaluation SPCP.

In relation to objective two, the barriers included: whether SPCP was a ‘real’ inter-municipal partnership; the clarity of SPCP’s vision/purpose; the ‘culture’ and ways of working of the partner agencies; leadership issues; and, the development of the budding relationships, links and connections that were being established by these new partners. These findings supported the conceptual model proposed by El Ansari & Phillips [17], which highlights partnership barriers of organization, of attitude, and of vision [21]. We explore below each barrier individually, although in reality they were all intricately meshed together and difficult to isolate.

As regards ‘real’ partnership, genuine partnerships require time, effort and resources. The groundwork preparation for a partnership [32], as well as stakeholder engagement and commitment could be arduous but critical for success. Partnerships and collaboration (shared risks in order to enhance capacity) are different from simple networking (exchange of information) and from coordination (shared activities and schedules). Collaboration builds on clarity and transparency in order to keep the partnership practical and operational [23]. Defined shared vision, mission, goals and transparent expectations underpin the action plans, objectives and time frames [32], resulting in more involved participants [32,33]. Indeed, clarity of purpose strengthens the stakeholders’ support for the partnership, keeping the effort dynamic, viable and effective. Since SPCP’s inception, the extent of collaboration, role clarity and expectations could all have benefitted from being clearer; and it would have been advantageous for the partners to have consensus and clarity as to the ‘mode’ of joint working they were operating on. Collectively, these findings supported the framework [17] that guided the present evaluation, which emphasized issues of membership, resources, support, processes, functions, and roles [18].

SPCP members felt that the ‘culture’ and ‘ways of doing business’ of the participating agencies were also barriers to success. Differences in stakeholders’ values are not uncommon in partnerships [17]; are key in deciding whether to partner or not; and may precipitate conflict [34]. It is critical that SPCP’s diverse partners are not ‘strange bedfellows’ in terms of differences in their values and ways of doing ‘business’. These findings fitted the framework [17] that we worked with, which explored the organizational characteristics and barriers that enable partnerships to fulfill their goals. [19].

Leadership across organizational boundaries has generally received little attention [1]. As regards SPCP’s leadership, successful partnerships are associated with good leadership [17], and partnerships that value their leaders have satisfied members with greater sense of ownership [11]. In collaborative efforts, skillful leadership makes things happen throughout the partnership’s ‘life cycle’: from development and formation, to implementation, maintenance, and institutionalization [35]. The lack of continuity of SPCP’s leadership was detrimental, where managers incrementally develop more fruitful relationships with stakeholders. These leadership finding resonate well with the conceptual model published by El Ansari & Phillips [17], where personnel and organizational factors and barriers play critical roles in successful partnerships [19].

With reference to the relationships, the links and new connections that were forged as a consequence of SPCP are important to the partners. Hence an understanding of partnership theory [17] and social network theory would be useful, although the general lack of theoretical frameworks for understanding partnerships is a barrier [35]. As stakeholders share responsibility for the processes, the possibilities to create fruitful relationships/connections with other actors are critical. This might not have been possible without SPCP’s stakeholders working as partners. Indeed as a result of SPCP, the municipalities started forging contacts with an independent boarding school for lower secondary students and one factory. Hence, viewing SPCP as a journey rather than a destination is constructive and valuable for the development of such links. This further supported the conceptual framework we employed [17], highlighting the political scientists’ emphasis on importance of ideology similarity in partnerships [20] in assisting these links to flourish and succeed.

The third objective was to provide recommendations on how the partnership can be further developed in order to advance SPCP’s aims. Based on our findings, we suggest the basis for an agenda for action for stakeholder individuals and organizations to include:

- Municipalities embarking on partnership efforts would benefit to initially explore the feasibility of such initiatives. Heterogeneities due to type of agencies and working methods requires early attention to develop fruitful partnerships in order not to miss opportunities for collaboration, experience exchange and the sharing of good practice. The time and resource challenges of creating a new structure (partnership) could be great, hence the economic support for such efforts should be appropriately gauged. Other national legislative or administrative reforms or local re-structuring that are implemented as the same time as the partnership effort should be considered in order to select an appropriate ‘time window’ of opportunity.
- Early attention and review of the clarity of the vision and mission of the partnership, where innovative and pragmatic approaches can be used to outline a realistic action plan and ensure that the participating organizations are ‘ready’ for the proposed interventions and goals. Clarity of the municipalities as regards the ‘level’ upon which they will collaborate is important.
- When training is provided to the partners in order to deliver certain interventions (e.g., a smoking cessation course), their organizations, agencies and departments need to satisfy the partnership that they are committed to such goals and would provide the time and administrative support for those stakeholders to deliver the task/s they were trained for. The ‘conditions’ in which the partners will be delivering certain interventions require thorough study in order to generate guidelines.
- The leadership issues that are encountered in partnership arrangements need special attention, as well as the continuity of such leadership over time. Leadership would include the actual leaders of the partnership as well as the leaders of the participating agencies that comprise the stakeholders.
- In future, it could be productive to recruit external ‘facilitators’ with track record in partnership working to review a partnership’s proposals before activities are started, and provide hands-on advise and support to the budding partners.

As regards the fourth objective, we assessed whether an example (SPCP) of ‘obligatory’ collaboration between municipalities in reference to smoking cessation intervention was at all feasible or desirable. In many instances, the availability of funding and resources act as stimuli for partnerships to be initiated and developed. This in itself need not be regarded as a disadvantage. However, a point to note is that a partnership (as a vehicle or intervention) is usually not employed as a first option to assist in solving public health and health promotion problems. Usually other solutions/interventions would have been implemented and would not have been successful in accomplishing the desired solutions. Hence, when partnerships are called upon as vehicles in health promotion, frequently the problems are then deeply entrenched and long standing; involving many stakeholders who might have traditionally not communicated or worked with each other; the solutions to the problems usually require behavioural change that requires time; and the problems would usually have exhibited resistance to other ‘solutions’ that had been tried in the past. In such situations, partnerships could be likened to national tertiary hospitals that usually receive nearly all the patients with serious, complicated and late conditions. Consequently, when such hospitals exhibit a higher mortality rate, that does not necessarily mean that their care provision is sub-optimal, but rather, it is a feature associated with the conditions of the patients they receive.

Given the pre-condition that the partnership was being established while processes related to the structural reforms were simultaneously being undertaken in both municipalities at the same time, testing the ‘success’ of the partnership was not an explicit aim of this research, as times of re-structuring and organisational reforms are viewed as sub-optimal opportunity windows for such evaluations. Nevertheless, such assessments of partnership working that we undertook are critical in order to provide formative feedback, guidance and lessons of good practice to such ‘budding’ collaborative efforts. However, it is reasonable to conclude that this partnership initiative faced many challenges that contributed to its low success. Indeed all the characteristics outlined above would need to be considered before a partnership is dismissed as a ‘failed experiment’. Further, given the availability of funding as a driver, agencies and organisations are frequently and hurriedly ‘pushed’ into partnership arrangements, where funding bodies would expect to be able to measure tangible results within a short time frame. Tight time frames do not do justice to the use of partnership approach in health promotion where behavioural change is usually an ultimate aim. In addition, initial training in partnership working is rarely provided to potential stakeholders as a preparation for their collaborative effort, and in the case of SPCP, training in partnership working *per se* was not provided at all. In health promotion, a common pitfall is that training is usually provided for the intervention (smoking cessation); but the vehicle of the intervention (partnership) itself is not viewed as a *bone fide* intervention that also merits experience, expertise and training. Partnership working is a science and art [2]. Hence in assessing whether the partnership was successful or not, collectively, these characteristics suggested that it is not merely the ‘obligatory’ nature of the collaboration between municipalities that is at hand, but a myriad of other factors need to be simultaneously considered. Thus the question: ‘was the partnership successful or not?’ is an important one. However it is difficult to divorce the question from a second question: ‘were the circumstances and conditions that are conducive for a successful partnership provided?’ Using El Ansari & Phillips conceptual model for partnerships [17] which we employed in the evaluation, SPCP would have greatly benefited from more thought about what successful partnerships really require and entail.

This study has limitations. It explored a partnership implemented in two particular municipalities, and hence caution needs to be exercised in generalizing the findings to other Danish municipalities or further afield. Furthermore, because of a change of SPCP’s leadership, the complete data about the smokers who participated in the smoking cessation course and those employees who were trained as smoking cessation consultants were not available. Such data, as an objective measure, would shed light about the effectiveness of the partnership. Similarly, the smoking cessation status was confirmed by the participants at the end of their smoking cessation course but was not independently bio-chemically validated, where such objective validation of the cessation status would have been useful. As our sample comprised 12 interviews, we were unable to indicate any individual participants’ role in SPCP in conjunction to their quotes, in order to preserve participants’ anonymity and confidentiality.

On the macro-level, future research needs to explore how concurrent government reforms interact with and affect budding partnership initiatives, and whether a ‘minimal time window’ is desirable between such reforms and subsequent partnership efforts. On the micro-level, research needs to address the specific impacts that are strongly and early affected by any lack of continuity in leadership in collaborative efforts. While the implementation of smoke-free environments was boosted by a legislation on smoke-free public places and workplaces, one may speculate that other macro level measure, especially those related to the reduction of the demand for and supply of tobacco as suggested by the WHO Framework Convention on Tobacco Control [36] would have further enhanced the mostly micro level implementation activities of SPCP.

## 5. Conclusions

Since the Ottawa Charter for Health Promotion [6] community action and intersectoral collaboration have been fundamental principles in health promotion practice. This study concludes that partnership practice would benefit from considering four factors that influence such efforts. The first factor is the clarity of the partnership: its mission, vision, purpose, working methods, benefits and costs as well as partners. Partnerships are likely to be successful if all partners are clear and transparent about the collaboration. Second, partnerships require taking the structural and cultural state of all partners into account. The third factor is the impact of the context where a partnership is initiated (e.g., economy and financial reforms and structural changes) on the development of the partnership. Finally the impact of personal/individual factors (e.g., personal engagement in the project; relationships, links and new connections) and leadership features on the partnership requires attention. With these four factors in place, partnership working could be more fruitful.

## Figures and Tables

**Table 1:** Government reforms in Denmark: changes in responsibilities.

1973–2006		2007–Present
**Macro**	**State**	

Legislative power Partition of tax money in negotiation with municipalities and counties about general grants		Legislative power Partition of tax money in negotiation with municipalities

**Meso**	**Counties**	**Regions**

Fifteen counties Power to levy taxes Hospital steering/treatment Chronic diseases Health promotion and preventions activities Ambulatory treatment Secondary education Rehabilitation		Five regions No power to levy taxes Hospital steering/treatment (somatic and psychological) Plan hospital structure and function Receive payments for hospital treatment from the municipalities

**Micro**	**Municipalities**	

Less power Small geographical areas (215 municipalities) Responsible for all social services (e.g., elderly care), welfare services (e.g., unemployment benefits) and education services (e.g., primary schools), except health care		More power Bigger geographical areas (98 municipalities) Same responsibilities in addition to secondary education (e.g., high schools) Responsibilities that were previously with the counties Extended health promotion and disease prevention Rehabilitation New responsibilities Establish health agreements between municipality and region about cooperation/coordination within health sector Patient education

**Table 2:** Challenges and opportunities of the Danish partnership.

Aspect	Description
Economy and Finances	SPCP received less economic support than was originally applied for but was obligated to accomplish activities described in the proposals
Reform and structural changes	A range of national structural reforms were being undertaken simultaneously with SPCP implementation
Use of partnership recourses; lack of coordination	Smoking consultants, who received their smoking cessation education through SPCP, were subsequently refused by the leaders in their department to use their time to conduct the courses and implement the training that they received
New Smoking Act	The new Act on smoking was helpful for SPCP, but on the other hand it made some activities unnecessary, e.g., the courses for smoking prevention
Clarity	Extent of the cooperation, expectations and role clarity were all unclear from SPCP’s inception
Different cultures and ways of working	Esbjerg and Varde municipalities had different workings methods and structure, which rendered the partnership between them challenging
Continuity of leadership	Change of the project leader as well as changes within the steering group (some person moved from Esbjerg and Varde and vice versa) contributed to a fragmented leadership
Relationships, links and new connections	SPCP provided opportunities to create new connections with other actors, e.g., boarding school and private factory.
Individual factors	Personal engagement and a dedicated group that leads the development of SPCP

SPCP: The Smoking Prevention and Cessation Partnership.
